# Computer-vision based automatic rider helmet violation detection and vehicle identification in Indian smart city scenarios using NVIDIA TAO toolkit and YOLOv8

**DOI:** 10.3389/frai.2025.1582257

**Published:** 2025-07-22

**Authors:** Uttam U. Deshpande, Goh Kah Ong Michael, Sufola Das Chagas Silva Araujo, Vaidehi Deshpande, Rudragoud Patil, Ramchandra Alias Ameet Chate, Varun R. Tandur, Supreet S. Goudar, Shreya Ingale, Vaishnavi Charantimath

**Affiliations:** ^1^Department of Electronics and Communication Engineering, KLS Gogte Institute of Technology, Karnataka, India; ^2^Center for Image and Vision Computing, COE for Artificial Intelligence, Faculty of Information Science and Technology (FIST), Multimedia University Jalan Ayer, Keroh Lama, 75450, Bukit Beruang, Melaka, Malaysia; ^3^Department of Computer Science and Engineering, Padre Conceição College of Engineering, Goa, India; ^4^Department of Computer Science and Engineering, KLS Gogte Institute of Technology, Karnataka, India; ^5^Department of MBA, KLS Gogte Institute of Technology, Karnataka, India

**Keywords:** traffic violations, deep learning, DetectNet, Resnet18, NVIDIA TAO, YOLOv8, OCR

## Abstract

Two-wheeler traffic offenses are a well-known fact about the Indian Road scenario. In addition to endangering the offenders, these offenses also endanger other commuters. Two-wheeler traffic violations can take many different forms, such as overloading, triple riding, and helmetless riding. Effective identification and enforcement strategies are necessary for these offenses since they pose a serious risk to public safety. Due to the inadequacy of traditional traffic monitoring and enforcement techniques, advanced technology-based solutions are now required. Deep learning-based systems have demonstrated significant promise in identifying and stopping such infractions in recent years. We propose a two-step deep learning approach that leverages the strengths of pre-trained object detection models to detect two-wheeler riders and specialized helmet classifiers to identify helmet wear status as well as detect number plates. In the first stage, we utilized a highly efficient, robust, and accurate object identification DetectNet (Model 1) framework developed by NVIDIA, and it uses the ResNet18 Convolutional Neural Network (CNN) architecture as part of the Transfer Learning Toolkit known as TAO (Train, Adapt, Optimize). The second stage demands accurate detection of a helmet on the identified rider and extracting numbers from the violator’s license plates using the OCR module in real time. We employed YOLOv8 (Model 2), a deep learning-based architecture that has proven effective in several applications involving object detection in real time. It predicts bounding boxes and class probabilities for objects within an image using a single neural network, making it a perfect choice for real-time applications like rider helmet violations detections and number plate processing. Due to a lack of publicly available traffic datasets, we created a custom dataset containing motorcycle rider images captured under complex scenarios for training and validating our models. Experimental analysis shows that our proposed two-step model achieved a promising helmet detection accuracy of 98.56% and a 97.6% number plate detection accuracy of persons not wearing helmets. The major objective of our proposed study is to enforce stringent traffic laws in real-time to decrease rider helmet violations.

## 1 Introduction

**Figure 1:**
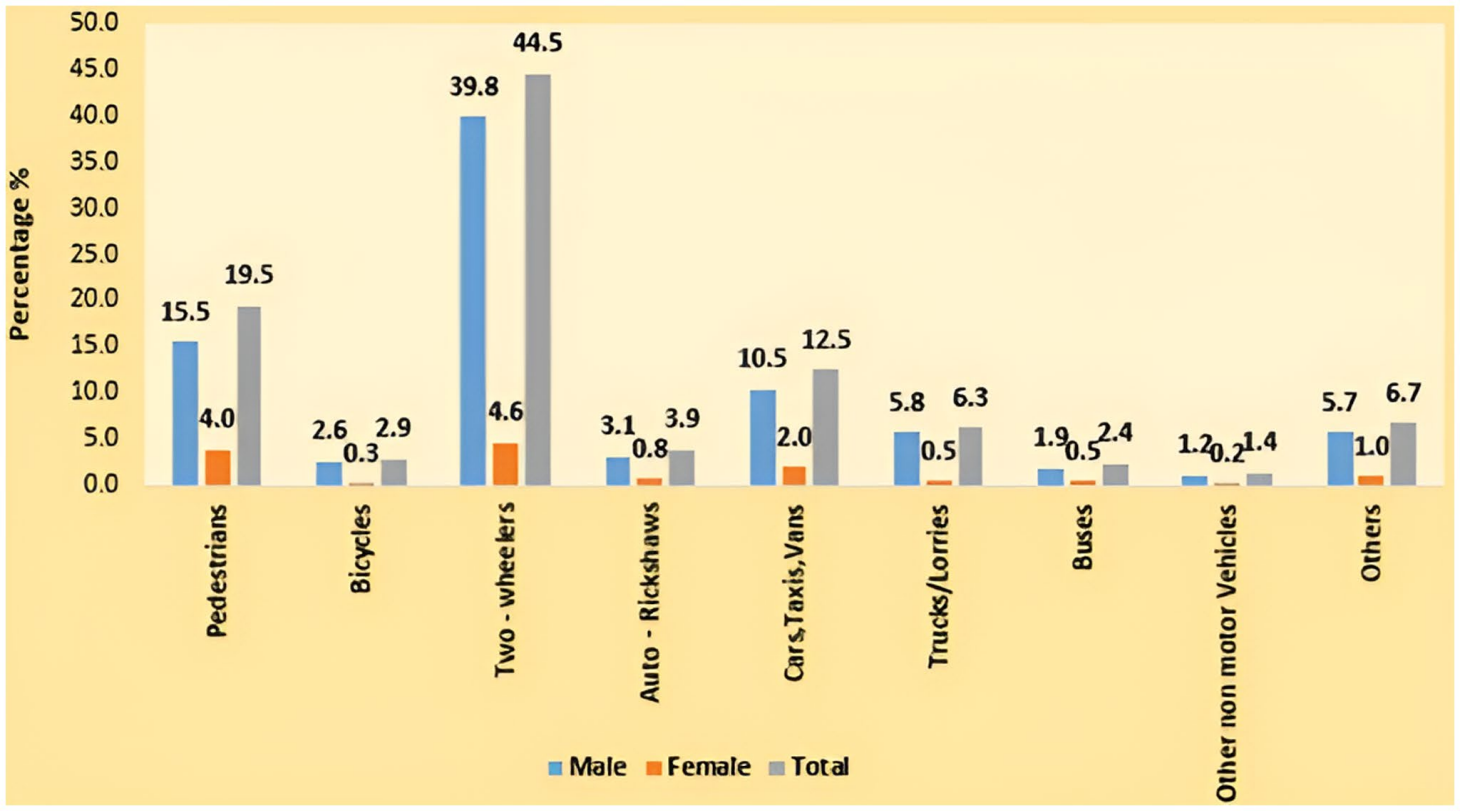
Road user groups and gender-specific profiles of those killed in traffic accidents (%) in 2022 (Road Accidents in India, 2022).

High rates of traffic infractions, especially from two-wheelers, can be commonly seen in Indian road scenarios. In addition to the cyclists themselves, other commuters on the road are also at risk from these violations. To solve this problem, deep learning-based detection algorithms that can precisely recognize and categorize various two-wheeler traffic offenses are in high demand. In India, two-wheelers are the most popular form of transportation, and they make up more than 78% of all vehicles on the road. Due to ignorance or a lack of safety concern, they frequently break traffic laws and are responsible for roughly 29% of traffic accidents (Road Accidents in India, 2022). A total of 74,897 people were killed in two-wheeler accidents, accounting for approximately 44.5% of all traffic fatalities according to a road accident report (Road Accidents in India, 2022). Two-wheelers accounted for the largest percentage (44.5%), followed by pedestrians (19.5%) and vehicles such as cars, cabs, and vans (12.5%), according to Figure 1. One of the main contributing factors to most two-wheeler accidents is the carelessness of motorcyclists who fail to wear helmets, which frequently leads to head injuries that cause trauma (Gopalakrishnan, 2012) to the skull or brain. 12% of motorcycle riders wearing helmets experience head injuries, compared to 20% of those not wearing them (Ravikumar, 2013). If there is a system in place to enforce helmet laws on motorists, they will be effective in reducing fatalities and injuries to the motorists. The number of people wearing helmets has increased after such laws requiring their usage have been imposed (Hagel et al., 2011). Manually monitoring traffic laws at every road and intersection in a city is very challenging and prone to errors. Thus, developing a real-time automated system that can identify and notify of traffic violations is crucial. These days, there is a lot of research being done on the detection and categorization of moving objects, which is largely utilized in applications like human motion capture and Intelligent Transportation Systems (ITS). Traffic monitoring and accident management are the two main ITS problems. It is undeniable that many traffic accidents are caused by careless and reckless motorbike riders. It is a need of the hour to have an automated system that uses computer vision to detect motorcycle riders who are not wearing helmets and punish them as per the law. As a result, traffic cops might have reduced the helmet violation monitoring burden. This can also lower the fatality rate, which has been sharply rising recently because of motorcycle accidents (Gopalakrishnan, 2012). Global research assessments demonstrated that the presence of surveillance cameras decreased the number of fatal accidents that resulted in serious injuries from 40 to 11% (Wilson et al., 2010). The development of an automated system that uses security cameras to automatically identify bikers who are not wearing helmets is therefore clearly vital.

We provide a deep learning-based method for identifying traffic violations involving two-wheeler vehicles that makes use of YOLOv8. It is a state-of-the-art object recognition model with great accuracy that can identify objects in real-time. Deep learning-based detection systems examine images and videos taken by security cameras positioned at key points along the highways using neural networks. Numerous traffic violations, including speeding, wrong-side driving, helmetless riding, and red-light jumping, can be identified and categorized by these systems. When it comes to detecting traffic violations, deep learning-based detection systems have a few advantages over conventional techniques. These algorithms have a high degree of accuracy and can identify even small infractions that human observers might miss. Additionally, these systems can run continuously around the clock, guaranteeing constant road surveillance. All commuters can benefit from increased road safety and a deterrent effect on offenses. To ascertain whether a violation has taken place, our system first detects the two-wheeler vehicle in the picture or video frame and then examines its behavior. To take further action, the device records the license plate number and follows the movement of the two-wheeler. The two functional modules of this suggested approach are helmet object detection and license plate recognition. The most effective YOLO neural networks are used in the object detection module.

In this research, we have generated the datasets by gathering pictures and videos of two-wheeler traffic infractions in the context of Indian roads. Bounding boxes and class labels were then added to the data to produce a labeled dataset that could be used to train the YOLOv8 model. Preprocessing was done on the dataset to guarantee consistency and noise elimination. To ascertain whether a violation has taken place, our system first detects the two-wheeler vehicle in the picture or video frame and then examines its behavior. To take further action, the device records the license plate number and follows the movement of the two-wheeler. The two functional modules of this suggested approach are helmet object detection and license plate recognition. The system effectively recognizes license plates in pictures, videos, or real-time by utilizing YOLOv8’s potent object detection capabilities. Accurate license plate recognition is therefore made possible by the application of OCR algorithms, which extract alphanumeric characters from the detected plates. By combining YOLOv8 and OCR, the system becomes more resilient to changing circumstances, which improves performance in practical situations. Our suggested YOLOv8 model provided a promising helmet recognition and number plate detection accuracy for individuals who were not wearing helmets. Our main goal is to lower the frequency of these traffic incidents by utilizing an automated computer vision method that identifies the two-wheeler rider’s helmet-wearing status. The results demonstrate that the proposed system can be beneficial to the traffic department in taking the appropriate measures against those who break traffic laws. Undoubtedly, this technique will promote a great deal of discipline in drivers to adhere to traffic laws. It will save the government resources needed to continuously monitor traffic infractions.

Summary of our Novel Contributions is as follows:

1. We developed a diverse dataset comprising 1,715 images, sourced from video footage captured using cameras positioned at various heights and angles across traffic junctions and intersections under varying lighting conditions.
2. We implemented an advanced transfer learning-based data preprocessing pipeline featuring a ResNet18-based DetectNet_v2 (Model 1) bounding box regressor to generate accurate boundary representations of two-wheelers.
3. We deployed a YOLOv8 (Model 2) to identify the number of riders, detect helmet violations, and extract vehicle information from complex traffic scenes. Experimental results demonstrate the model’s effectiveness in detecting multiple objects across large field-of-view traffic environments.

The remainder of the paper is organized as follows: A literature review is provided in Section 2. The proposed helmet violation detection technique is presented in Section 3. Section 4 covers the experimental findings and observations. Section 5 finally concludes the work.

## 2 Literature survey

Computer vision techniques are increasingly being applied to the investigation of helmet wear compliance these days. It entails determining if motorcycle riders are wearing helmets while riding. Digital image processing and computer vision methods are utilized in many object recognition applications, including autonomous driving, automatic rider helmets, pose detection, and video surveillance applications (Buch et al., 2011; Vargas et al., 2010; Buch et al., 2010; Yang, 2022; Ashiq et al., 2022). These strategies can be broadly divided into two categories: deep learning techniques and machine learning techniques.

### 2.1 Machine learning-based approaches

There are chances that a person may get confused between a safety helmet and with human head because of their similar shapes. John et al. suggested a method for detecting motorcycles and tracking their movements over time that makes use of Histogram Oriented Gradient (HOG) features (Chiverton, 2012). The technology determines if a helmet is present by analyzing the corresponding rider region after detecting a motorcycle. Rattapoom et al. suggested a four-step procedure (Rattapoom Waranusast et al., 2013) that recognizes the presence of a motorcycle and ultimately categorizes every individual on it to automatically identify motorcycle riders and ascertain if they are wearing head protection. Three features are extracted by the system after separating moving and stationary objects: the area of the bounding rectangle containing the image, the aspect ratio of the rectangle’s width to height, and the standard deviation of the hue surrounding a rectangle at the object’s center. To determine whether the object is a motorcycle or another moving object, the K-Nearest Neighbor (KNN) classifier is applied to the three features that have been extracted from the moving object. To identify bikers without helmets, Silva et al. developed a hybrid descriptor model based on texture and geometry data (Silva et al., 2013). To identify the biker’s head, SVM and the Hough transform (HT) are employed. The algorithm has been trained using a self-generated dataset. To distinguish between various items, they expanded their research and employed a multilayer perception model, achieving an accuracy of 94.23%. To assist in extracting the properties of the image, they also suggested a technique based on HT and histogram-oriented gradient (HOG) (Silva et al., 2014). The roadside cameras provide the input image database of 255 samples, and the system produced helmet detection accuracy of 91.37%. A technique based on the K-nearest neighbor (KNN) classifier was proposed by Waranusast et al. to identify and detect motorcycle riders wearing and not wearing helmets (Waranusast et al., 2013). The self-generated dataset has been used to test the system, and the images were captured using a webcam. According to the trial results, 68, 84, and 74% of the far lane, the close lane, and both lanes, respectively, were successfully detected by the system. A technique that uses a Histogram Oriented Gradient (HOG) to extract characteristics is used to identify motorcycles and monitor their movements over time. To ascertain whether a helmet is present, the system examines the matching rider region after detecting a motorcycle. This is accomplished by employing a support vector machine (SVM) classifier that was trained using histograms from the motorcyclists’ head region image data, which were calculated using the HOG descriptor (Aboah et al., 2023; Armstrong Aboah et al., 2023). On the other hand, these methods do not identify or count the number of motorbike riders.

### 2.2 Deep learning-based approaches

More recently, effective Deep Learning methods have been developed to precisely identify the presence of a motorcycle and the rider. To find objects in motion, Vishnu et al. employed an adaptive search technique (Vishnu et al., 2017). Following that, bikes were distinguished from objects in motion using a CNN on a self-generated dataset. Lastly, CNN is used to distinguish between bikers who are not wearing helmets. Mistry et al. detected helmetless bikers using CNN (Mistry et al., 2017). YOLOv2 was utilized at two levels. First, the system detected various objects and helmetless motorcycle riders using YOLOv2. For training, the COCO dataset has been utilized, and experimental results reveal an accuracy of 92.87%. Raj et al. used a deep learning technique (Raj et al., 2018) to help identify bikers who had flouted rider helmet regulations. HOG is used for motorbike detection, and the region of interest is chosen after that. To recognize number plates and identify bikers without helmets, they used CNN technology. Self-generated data from various sources has been utilized, and 94.70% accuracy has been reported. A deep learning method called RatinaNet50 was used by Siebert and Lin (2020) to identify bikers who were not wearing helmets. Self-generated data was utilized to train the suggested system. There are now two classes: With-Helmet and Without-Helmet. The experimental result shows an accuracy of 72.8%. Faster R-CNN (Afzal et al., 2021) was used by Afzal et al. to identify motorcycle riders who had not worn helmets. Custom-generated data was used to train the system. According to the experimental data, the accuracy reported was 97.26%. Using deep learning techniques based on the YOLOv4 model (Kharade et al., 2021), Kharade et al. presented a system for identifying motorcycle riders who are not wearing helmets. When compared to existing CNN-based algorithms, the suggested approach shows true performance in traffic motion images.

The YOLOv3 algorithm (Kathane et al., 2022) was implemented by Kathane et al. Advanced deep learning models are trained to recognize objects. Three distinct deep learning models are used by the developed system to identify these things. The established technique provides 91.8% precision for number plate detection and 88.5% precision for motorcycle detection. Using a convolutional neural network (CNN), Rajalakshmi and Saravanan (Rajalakshmi and Saravanan, 2022) created a system for tracking and dealing with those who violate the rules. Using a suitable CNN-based model, the system carries out vehicle classification, helmet detection, and mask detection. Although the methods can identify motorcyclists who are not wearing helmets, they have certain drawbacks. The accuracy of most current systems is low. Furthermore, there is a restricted dataset that was utilized to construct the system. Furthermore, a few of the systems mentioned above are unable to distinguish between a scarf and a helmet. YOLOv5 is a deep learning-based object identification system with real-time object detection capabilities. The design is based on the same methodology as YOLOv3 and YOLOv4, but it incorporates a few enhancements to increase its accuracy and efficiency. YOLOv5 is based on a modified version of the EfficientNet architecture, which is renowned for its accuracy and efficiency. CSPNet, the YOLOv5 backbone, is made up of a bottleneck block that lowers the number of channels and several convolutional layers. To improve road safety and lower accident rates, YOLOv5 is being used for the real-time detection of two-wheeler traffic offenses in Indian road settings. Tasbeeha et al. built a real-time system that uses Faster R-CNN (Waris et al., 2022) to recognize motorcycle riders without helmets in roadside camera surveillance footage. For effective helmet violation detection, the algorithm makes use of a Fast R-CNN and a region proposal network (RPN). To provide a variety of settings, the dataset was created by combining pre-existing datasets with self-captured pictures from Lahore, Pakistan. In experimental analysis, the system outperforms competitors in helmet violation detection with a high accuracy of 97.69%. Although the system performs better thanks to its sophisticated algorithm and large dataset, it is unable to recognize violators’ license plates.

Agorku et al., utilized YOLOv5 and ensemble learning (Agorku et al., 2023) to develop a real-time helmet violation detection system to improve the identification of motorbike riders and passengers and determine if they are wearing helmets. Five models with various hyperparameters are used in an ensemble learning strategy to improve YOLOv5’s object identification capabilities. The suggested model achieved a mAP score of 0.5267, and the precision/recall values of the model can be increased by training on a wider variety of datasets. The ResNet18-based DetectNet_v2 model (Deshpande et al., 2025) was deployed to reliably predict triple riding on a two-wheeler and produced triple-rider detection accuracy of 91.42% under challenging environments. The combination of dense layers of ResNet-50 to perform image classification tasks and YOLOv8 for real-time object detection (Deshpande et al., 2025) to accurately detect small objects like cell phone usage produced a Mean Average Precision (mAP50) of 49.5% at 0.5 IoU for detection tasks and an accuracy of 96.03% for prediction tasks. As mentioned above, most of these literatures lack systems with real-time rider identifying capabilities, separating passengers from the motorists, and determining if a helmet is worn. Furthermore, these models do not extract vehicle number plates, making it difficult to identify violators. Our proposed research introduces a cutting-edge real-time model that can identify motorbike helmets and their number plates in smart city scenarios.

## 3 Proposed methodology

The major challenges faced in carrying out research in rider helmet detection are the lack of a publicly available dataset. While we explored popular platforms like Kaggle and COCO, we encountered limitations in finding images that met our specific requirements, including complex backgrounds and diverse lighting conditions. Our first challenge revolved around collecting a suitable dataset for training our helmet detection model, and we successfully overcame this by generating a custom dataset. This section outlines the development of a custom dataset from surveillance cameras positioned along the roads, intersections, traffic junctions, and captured in sunny, cloudy, rainy weather at daytime, dusk, and nighttime lighting conditions.

**Figure 2:**
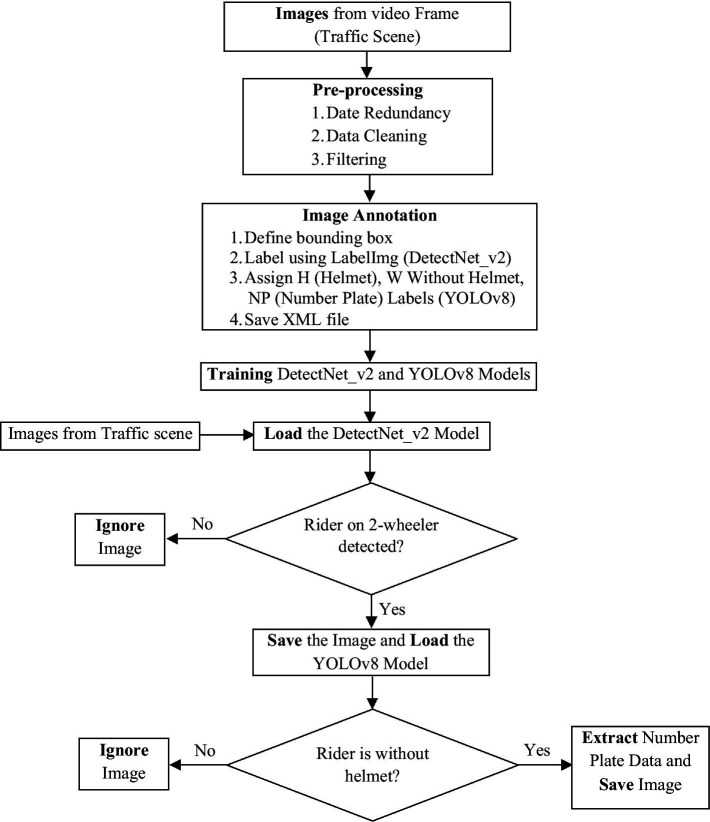
Block diagram of the proposed two-step helmet violation detection system.

We proposed a two-step system to identify riders on a two-wheeler and detect persons not wearing helmets, including their license plates. In the first stage, we utilize DetectNet_v2 as Model 1 to accurately identify individuals riding bikes/scooters on streets and junctions. This model is trained on the generated ground truth images to distinguish riders from others like four-wheelers, bicycles, and pedestrians, ensuring precise detection even in complex traffic environments with varying lighting conditions and vehicle densities. After the rider has been identified, the second stage entails applying the YOLOv8 as our Model 2, especially for helmet and number plate detection. When there is more than one rider on a two-wheeler, the model also displays the number of riders with their helmet wear status. Lastly, the system records the offending bike’s license plate, allowing law enforcement to carry out the necessary legal actions. Figure 2 illustrates the proposed two-step helmet violation detection system flow diagram. The various aspects of the proposed technique are explained in the following sections.

### 3.1 Data set collection, pre-processing, and annotation

The key to success in deep learning research, especially for object identification models like DetectNet and YOLOv8, is the availability of high-quality data. The more varied and extensive the data, the models can generalize their understanding and function well in unknown settings. The data collection process began by physically gathering images from video footage obtained from a variety of traffic intersections from a range of perspectives and in varying lighting conditions.

The following crucial phases are involved in image extraction from a live video feed:

1. *Processing Video*: The cameras’ live video feed is recorded in this step.
2. *Identification of the Frame*: The frames from the earlier step are extracted, pre-processed, and then fed into deep learning models. The frames are extracted at 10 Frames Per Second (FPS).
3. *Different Heights and Angles*: Cameras were placed at various heights (10 to 15 feet) and angles to accurately represent the actual traffic scenes. This was done to ensure that the dataset could contain a variety of real-world camera locations and viewpoints.
4. *Diverse light conditions:* By collecting data in a range of lighting situations, we ensured that the dataset could faithfully represent the differences in visibility and appearance of traffic scenarios during the day, night, cloudy, and rainy lighting scenarios.

To create an extensive data set for our study, images were created by employing effective synthesis techniques to create separate images from video frames. We gathered 1,585 images from these video frames and added another 130 images from the online COCO dataset, making them total of 1,715 images containing two-wheeler rider images with and without helmets, as summarized in Table 1. After a rigorous cleaning process to ensure data quality and relevance, 1,200 high-quality images of single, double riders with and without helmets were added to the final dataset.

**Table 1:** Custom dataset classification.

Dataset source	Riders with helmets	Riders without helmets	Total images
Acquired images	1,040	545	1,585
Online dataset (COCO)	85	45	130
Class-wise total images	1,125	590	1,715

**Figure 3:**
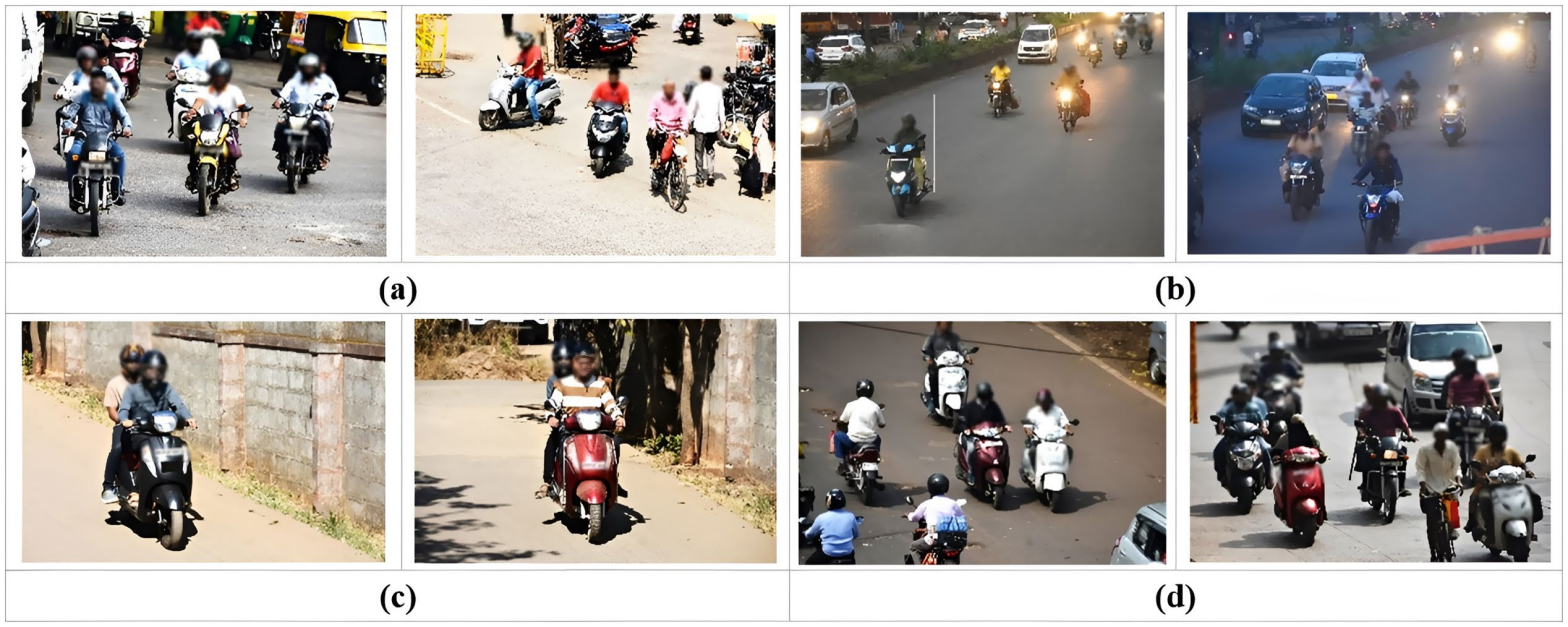
Images captured at different light conditions **(a)** day images, **(b)** night images, **(c)** synthetically generated images, and **(d)** complex scenario with multiple vehicles in opposite directions.

As seen in Figure 3, the captured images represent various urban scenarios depicting real-world helmet use cases. These environments included streets, intersections, and traffic lights. The aim was to capture data under a wide range of conditions, fostering a model that can adapt to real-world complexities. To make the dataset diverse, the images are captured in sunny, cloudy, rainy weather at daytime, dusk, and night-time lighting conditions. This ensures the model can handle variations in lighting and visibility that may affect helmet detection, as shown in Figures 3a,b. We also synthetically generated images with the help of various team members riding different two-wheelers under different road and lighting conditions as indicated in Figure 3c. Further, we captured images in locations with multiple objects at different complex backgrounds and vehicles moving in opposite directions, as seen in Figure 3d This helps the model to learn the helmet object from the surrounding environment, improving detection accuracy across various settings.

#### 3.1.1 Data set pre-processing and annotation

**Figure 4:**
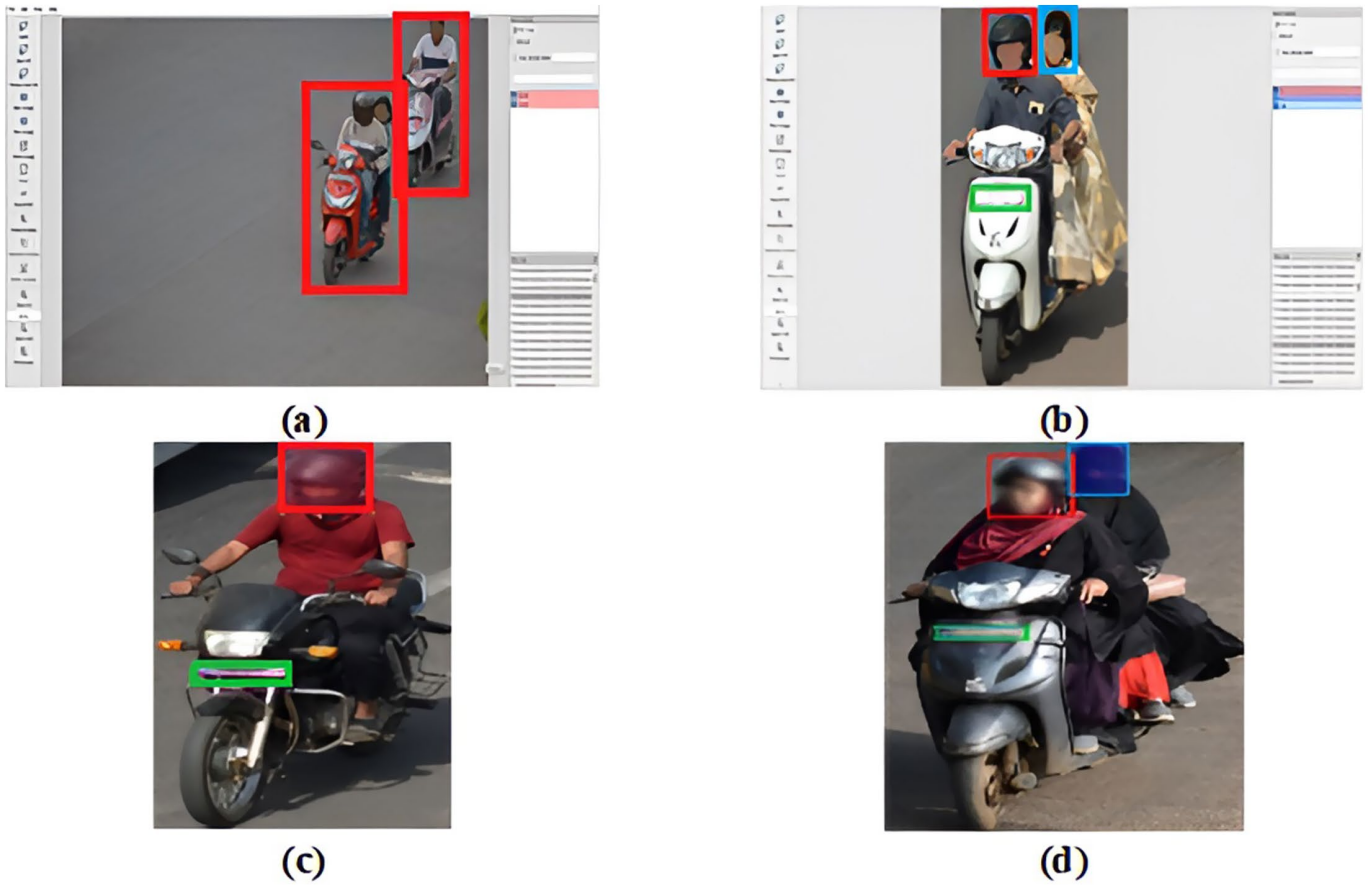
Sample annotated images. **(a)** Rider marked with Red is used for training DirectNet_v2, and **(b–d)** riders marked in red (with helmet), blue (without helmet), and green (number plate) for training YOLOv8.

It is necessary to preprocess the dataset to obtain the relevant data to perform further tasks. To eliminate missing objects, frames with irrelevant pictures, redundant data, etc., the dataset is manually pre-processed to choose relevant frames. These 1,200 images underwent data cleansing and filtering to eliminate any low-quality or irrelevant samples that included objects with unclear information due to poor lighting. After preprocessing, 1,107 images with riders are selected to train the DetectNet_v2 model. Similarly, a total of 1,071 images of riders wearing helmets, not wearing helmets, and mixed instances in different lighting conditions are identified to train the YOLOv8 model. An annotation task for image labeling has been carried out using the LabelImg tool (Labelimg, 2025). A bounding box is drawn around the image to assign four different labels. A label called “rider” is assigned to an image containing persons on a two-wheeler, as indicated by the red bounding box in Figure 4a. Images of bikers with helmets represent the “H” label and are marked with a red bounding box. Meanwhile, the “W” label indicates bikers without helmets and is marked with a blue bounding box, as shown in Figures 4b,d. The two-wheeler number plates in images are labeled as “NP” and are marked with a green bounding box as seen in Figures 4b,c.

To deal with overlapping motorcycle rider incidents found in a single image are properly delineated to provide comprehensive annotation results. The annotated metadata of images is first saved as .xml files (PASCAL or VOC) before being moved to the KITTI file format. This usually provides the location and attributes of the motorbike objects in the pictures, including their labels and bounding boxes (xmin, ymin, xmax, ymax). The regions of the recognized rider two-wheeler are output as bounding box coordinates by DetectNet after training is successful. This data is used as the input for the next phase.

### 3.2 Model selection

The DetectNet_v2 is trained after the dataset is prepared so that real-time and accurate two-wheeler riders can be localized from a complex traffic scene. This rider information is then sent to the subsequent YOLOv8 model to predict the rider helmet violations and penalize motorcyclists based on the information from the processed license plate.

#### 3.2.1 Rider localization using DetectNet_v2 (model 1)

**Figure 5:**
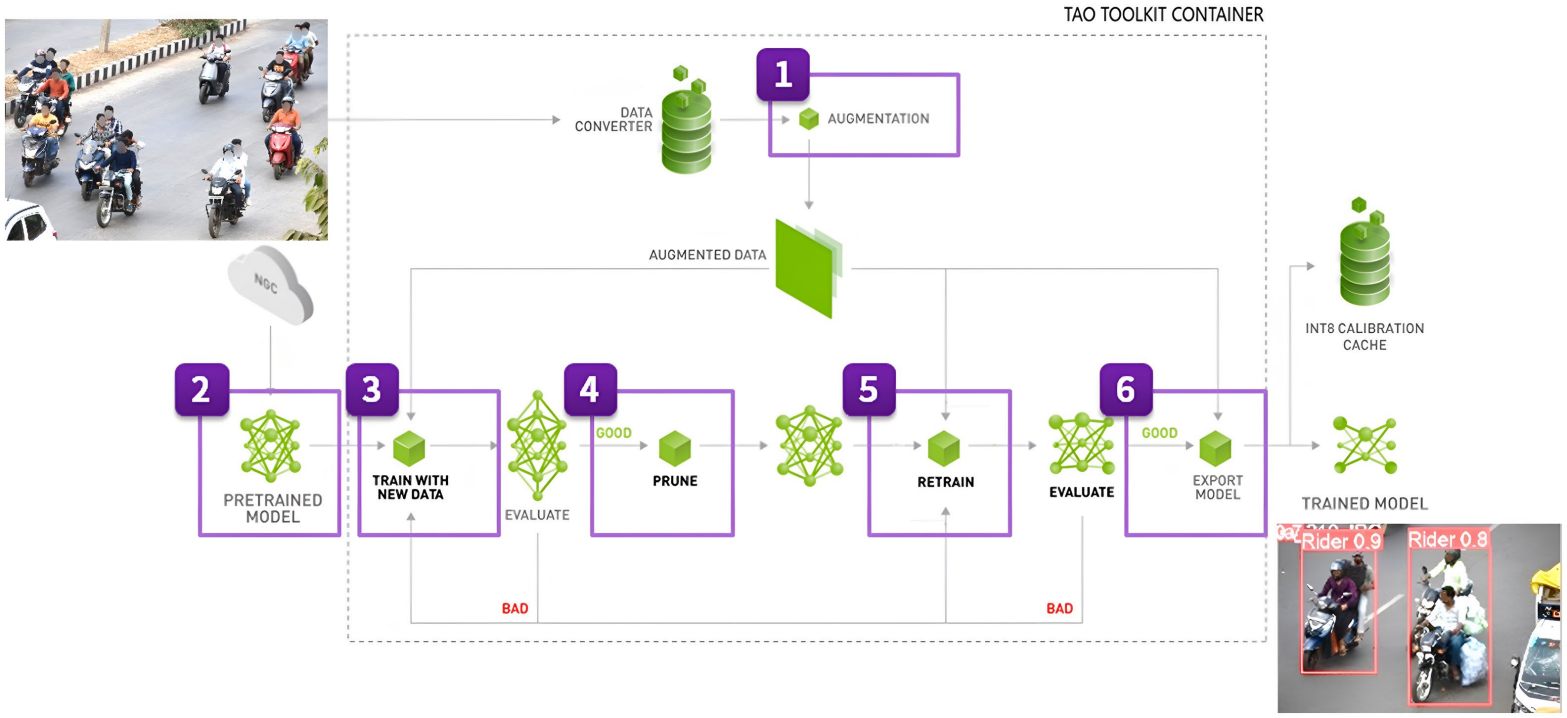
Rider detection and localization pipeline using TAO DetectNet_v2 (NVIDIA Documentation Hub, 2025).

In automated surveillance and traffic monitoring systems, identifying and distinguishing two-wheeler riders from other vehicles in traffic scenes is an essential challenge. For fast, real-time item localization and detection of objects in pictures or video streams, NVIDIA developed DetectNet, a sophisticated object recognition framework. As seen in Figure 5, it is an essential component of the TAO Toolkit, a set of tools created to make deep neural network design and optimization easier. Convolutional neural networks (CNNs) here are used to detect objects with a high degree of efficiency and accuracy, and it is built on the concepts of deep learning. The process of transferring the learnt features from one application to another is known as “transfer learning.”

**Figure 6:**
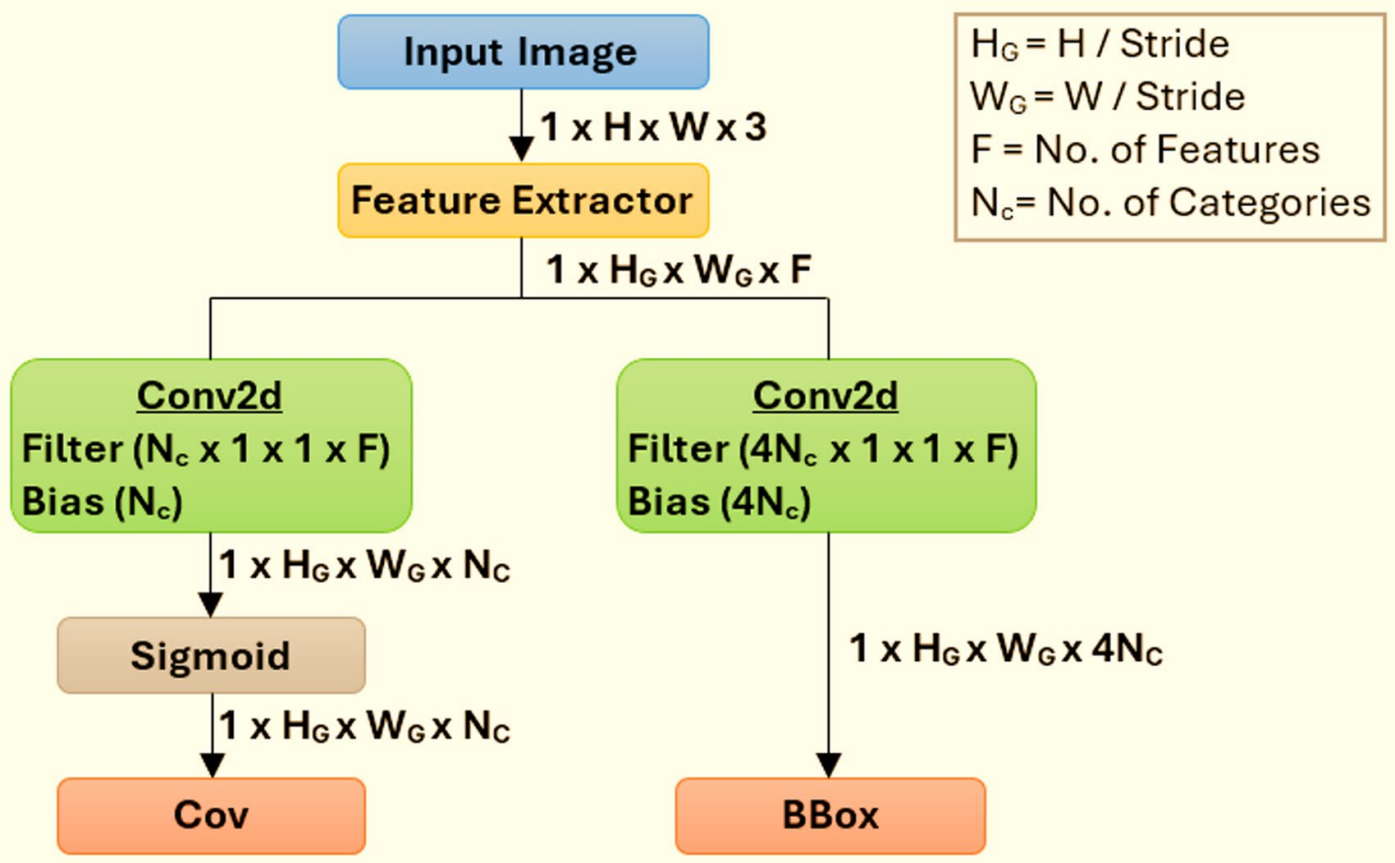
High-level architecture details of the DetectNet_v2 model.

We use DetectNet with pre-trained models that are tuned for object detection and use transfer learning techniques to refine them using task-specific data. Using a model that has been trained on one task and retraining it to utilize it on another is a popular training method. DetectNet can simultaneously predict class probabilities and object bounding boxes in a single network run due to its single-stage architecture, as indicated in Figure 6. To leverage the transfer learning to effectively recognize two-wheeler riders, we configure NVIDIA’s pretrained model that is part of TAO Toolkit (NVIDIA Documentation Hub, 2025), called “TrafficCamNet.” This model is built using DetectNet_v2 with ResNet18 as the backbone and trained on a subset of the Google Open Images Dataset (11,000 Two-Wheelers images). It is designed specifically for traffic scene understanding, and it refines its pre-trained convolutional layers to identify motorcycles and riders in urban traffic scenes using inputs from the KITTI dataset, such as labels and bounding boxes in the format (xmin, ymin, xmax, ymax). The detecting head adjusts to the unique geometry and look of two-wheeler riders, while the pre-trained backbone records general traffic object information. This method increases accuracy and speeds up training, especially when using a small custom dataset like our custom-developed dataset.

Figure 6 illustrates how DetectNet_v2 uses a GridBox architecture to divide the input into a uniform grid and a regression technique to forecast a confidence value (cov) and bounding box (bbox) for every category. This GridBox approach guarantees that every grid cell can identify an object and forecast its spatial bounds and class likelihood. Each predicted bounding box is given a confidence score by the model, which considers both the likelihood that an object would be present and its classification. The Gridbox system divides an input image into a grid to forecast four normalized bounding-box parameters, namely “xc,” “yc,” “w,” and “h,” together with the confidence value for each output class. Post-processing methods like Non-Maximum Suppression (NMS) are used to produce final bounding boxes and class labels using post-processing techniques like Non-Maximum Suppression (NMS) clustering after the model’s output has been scaled and the offset has been established. By keeping only the boxes with the highest confidence scores for each object observed, NMS assists in removing overlapping boxes. As a result, class labels and bounding boxes are accurate and unique.

The degree to which the predicted bounding box closely matches an actual object in the image is indicated by the confidence score in Equation 1.


(1) Confidence=Pr(Object)∗IoU

Two bounding boxes intersecting their union is represented by the Intersection of Union (IoU). It is obtained using Equation 2,


(2) $$\mathrm{IoU}=\frac{\text{Area of Intersection ofboxes}}{\text{Area of Union of}\;\mathrm{two}\;\text{boxes}}$$

The bounding box with the highest confidence level is retained, while the others are disregarded. To provide thorough traffic rule enforcement, the refined output—detected bounding boxes with corresponding confidence values—is used as input for further modules like helmet detection and number plate localization.

The model is tested with the following pre-trained parameters:

Input image (W × H × C) = Typically resized to 960 × 544 × 3 (Width × Height × Channels).

Input Channel Ordering (N × C × H × W) = Set N as 8 (Batch Size) and Number of epochs = 25.

Output: Confidence Scores (Floating point values), Bounding Box Coordinates (X, Y), Width (W), Height (H), and Labels (Text).

**Figure 7:**
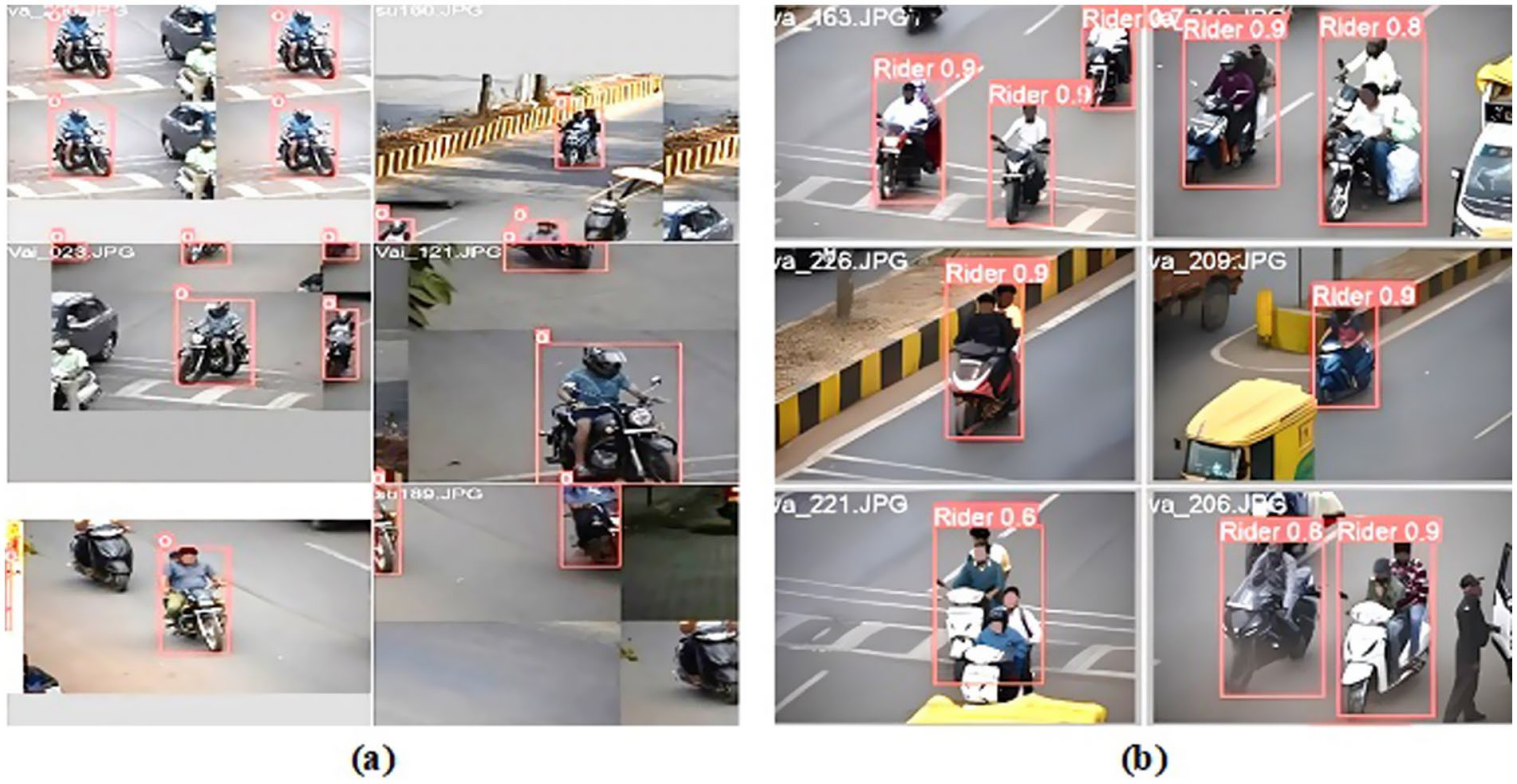
Proposed DetectNet_v2 (Model 1) two-wheeler rider detection system. **(a)** Rider localization. **(b)** Rider with confidence scores.

Figure 7 demonstrates the performance of the proposed DetectNet_v2 (Model 1) two-wheeler rider detection system. The rider detection is indicated by the label ‘0’ above red bounding boxes (see Figure 7a), and the two-wheeler rider with its confidence score is indicated by a red bounding box as shown in Figure 7b). Our proposed DetectNet_v2 model detects the actual two-wheeler riders with a high confidence score by ignoring other non-riders like vans and auto rickshaws correctly, as indicated in Figure 7b. This model uses deep learning techniques and can detect multiple riders simultaneously with high precision. This was the motivation for us to choose DetectNet as Model 1 in the detection pipeline for accurate rider detection compared to YOLOv8. The details are provided in the ablation study carried out in section 4.3. For rider helmet and number plate detection, we preferred the YOLOv8 as our Model 2 due to its low processing power and real-time performance. Our method is known as a two-step detection system for this reason.

#### 3.2.2 Helmet and number plate localization and detection using YOLOv8 (model 2)

**Figure 8:**
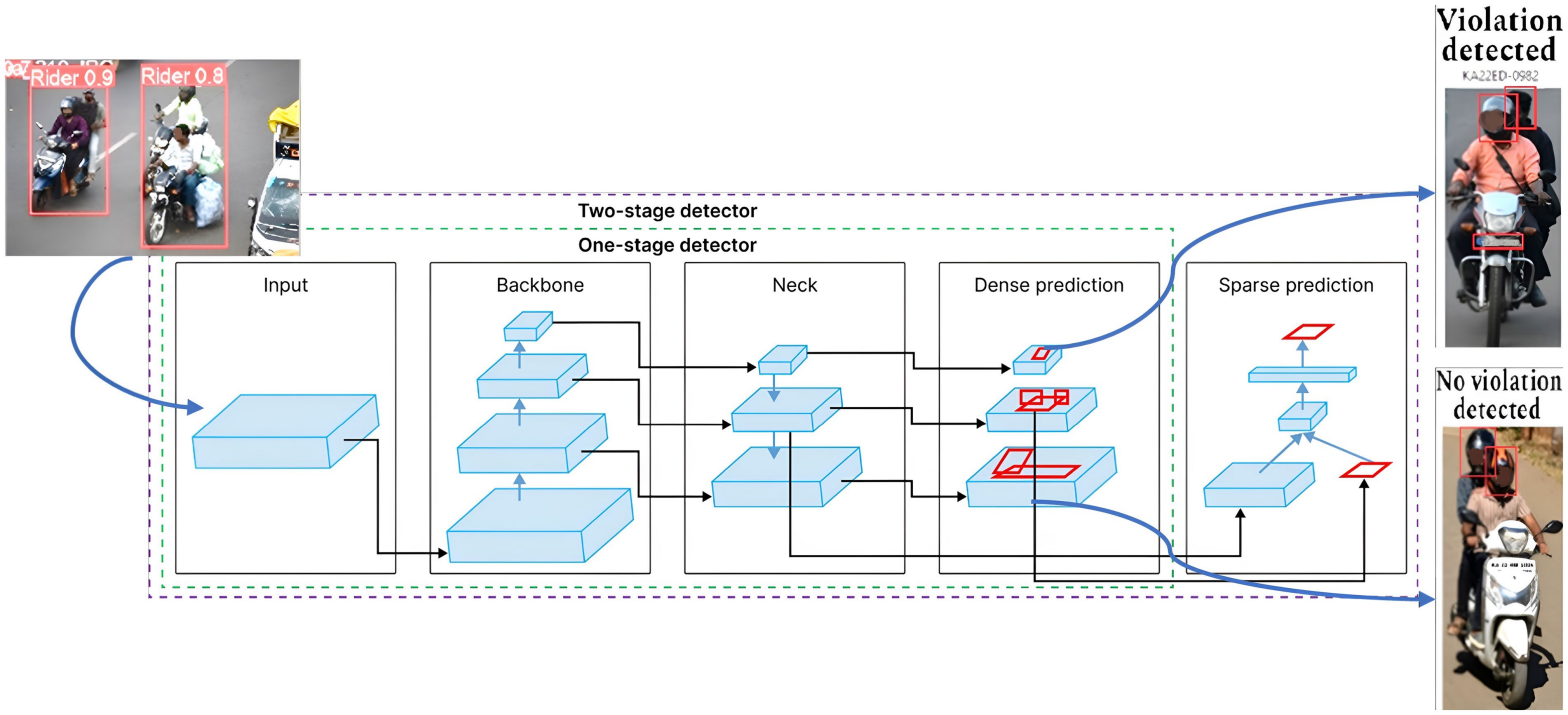
One-stage and two-stage object detector pipeline (YOLOv8: A Complete Guide, 2025).

You Only Look Once (YOLO) or Single Shot Detector (SSD) is a state-of-the-art, real-time object detection algorithm. It was introduced in 2015 by Joseph Redmon, Santosh Divvala, Ross Girshick, and Ali Farhadi (Redmon et al., 2016). What sets YOLO apart is that it tackles the object recognition problem as a regression issue rather than a classification task. For tasks involving accurate object identification, segmentation, and classification, this model is the most comprehensive architectural design that is currently in use. We use the YOLOv8n nano form of the YOLOv8 family that is pre-trained on the COCO dataset, which contains 80 object classes from diverse scenarios. This framework receives the two-wheeler rider image with bounding box values and labels produced by DetectNet_v2 as input, to accurately detect the riders and helmet wear status. Finally, the owner information is extracted through the OCR number plate extraction method that is inbuilt into the YOLOv8 architecture. Figure 8 provides an illustration of the YOLOv8 architecture for rider identification and triple-riding detection. The accuracy of single-pass YOLO architecture is often moderate when compared to two-stage detectors, but they are computationally less demanding and produce higher frame speed and detection speed.

In addition, all YOLO models share the same structures in their architecture, and are described below:

*Input*: The mosaic data augmentation plays a vital role in enhancing the functionality of computer vision models. First, input images are enhanced through the use of mosaic augmentation, which changes training by merging many images and improves the generalization and robustness of the model.

*Backbone*: The backbone is the workhorse of feature extraction. The backbone is a pre-trained Convolutional Neural Network (CNN) that extracts low, medium, and high-level feature maps from an input image. It is a convolutional neural network that generates and stores variously shaped and sized visual features. In contrast to conventional methods, this uses spatially distinct bounding boxes and assigns a probability to each detected object within a single convolutional neural network (CNN). In the context of CNNs, convolution is the process of slicing a filter, also called a kernel, over an input picture to create a feature map. As it passes over the input image, this filter records the spatial relationships between pixels by adding up the outcomes of element-wise multiplication. Typically, the convolution of two functions, such as (𝑡) and 𝑔(𝑡), is expressed as (𝑓∗𝑔) (t). The CNN convolution process is explained below with the help of Equation 3:


(3) $$(f*g)(t)=\int_{-\infty }^{\infty }f(t-\tau )\mathrm{d\tau}$$

Here, (𝑡) stands for the input image, and 𝑔(𝑡) the kernel or filter. The variable 𝑡 denotes a location in the output feature map where the output is evaluated. The CNN design, which consists of many convolutional layers and max-pooling layers, deepens feature maps while progressively reducing the input’s spatial dimensions. To process the collected information and generate predictions, YOLO incorporates fully linked layers, also known as thick layers, after the network. ResNet, VGG, and EfficientNet are examples of classification models that are employed as feature extractors.

*Neck:* The neck takes the feature maps extracted by the backbone at different scales. It comprises a group of layers that combine and integrate characteristics before sending them to the prediction layer. Path Aggregation Network (PAN) processes the feature maps extracted by the backbone at different scales (spatial resolutions). PANet cleverly merges these feature maps, creating a “feature pyramid” with rich spatial (capturing local details) and semantic (understanding object classes) information.

*Prediction head:* This final stage takes the processed feature pyramid from the neck and generates the final predictions. The head uses the bounding box predictions in addition to the neck’s features. It has three branches, and each branch has its feature scale. Three grid cells—13×13 (big object), 26×26 (medium object), and 52×52 (small object)—are used to generate a bounding box, class probabilities, and confidence ratings. Each grid cell predicts three bounding boxes. Finally, the network uses NMS to filter out overlapping bounding boxes. Anchor boxes are fixed-sized bounding boxes used to forecast the size and position of objects in a photograph. Instead of forecasting arbitrary bounding boxes for each instance of an object, the model uses established aspect ratios and scales to predict the anchor box locations. After that, these coordinates are modified to match the object instance. To provide efficient multi-scale object detection, the YOLOv8 neural network architecture combines the Path Aggregation Network (PAN) with the Feature Pyramid Network (FPN). To create feature maps that are sensitive to objects of different sizes, the FPN gradually decreases the input image’s spatial resolution while deepening the feature channels. The model can extract low-level data from shallower layers and high-level semantic features from higher ones because of its hierarchical structure. In addition, the PAN module improves the flow of localization signals from shallow layers to deeper layers by introducing bottom-up path augmentation via skip connections. By combining features from several network hierarchy levels, this combination gives YOLOv8 can detect objects of various sizes and shapes with greater accuracy.

**Figure 9:**
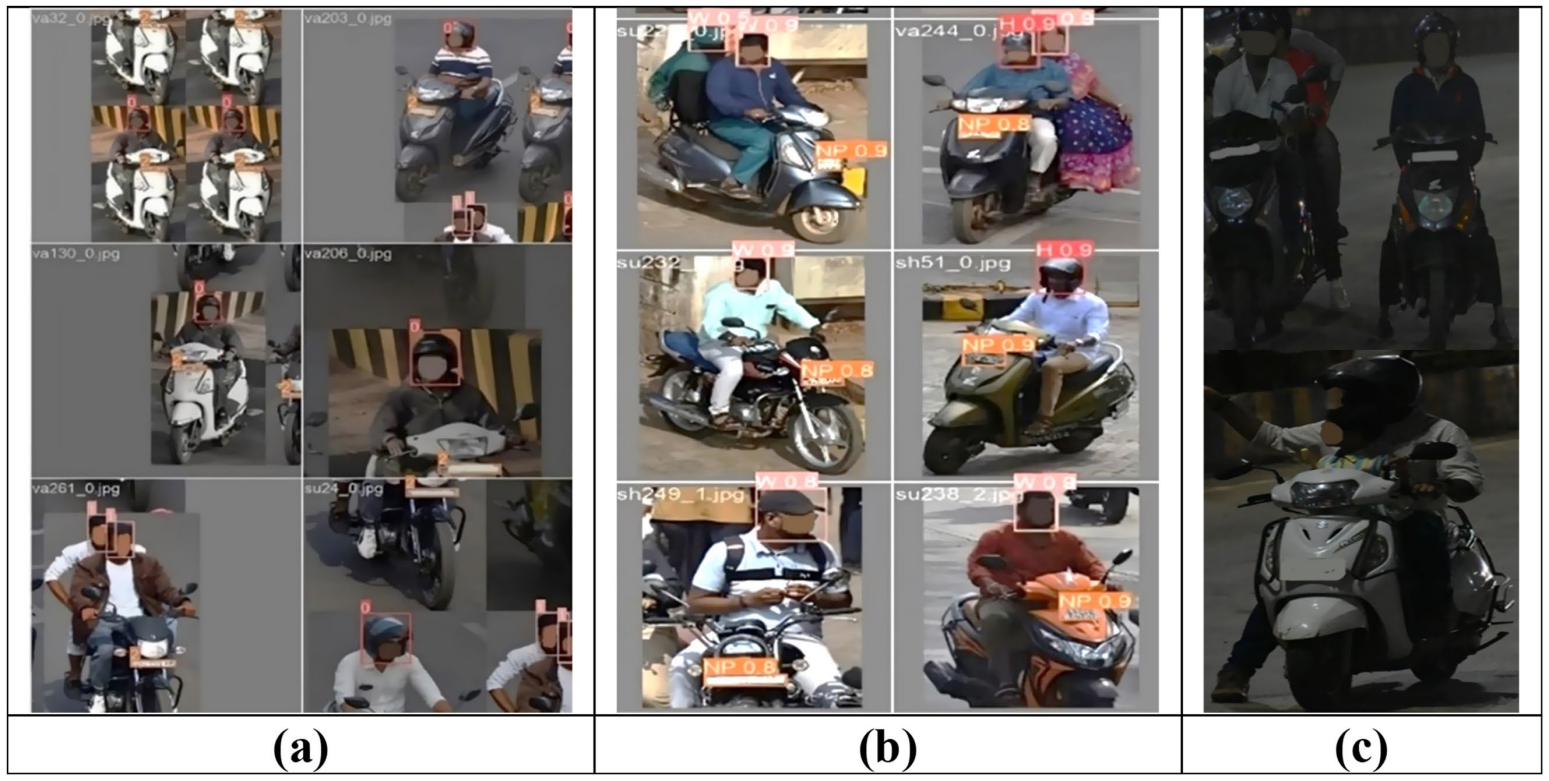
YOLOv8 helmet and number plate detection system performance. **(a)** Localization. **(b)** Detection with confidence score. **(c)** Few failure cases observed in low-light conditions.

The YOLOv8 model uses target regression on spatially separated bounding boxes to achieve excellent detection accuracy. The input image is first divided into a grid of N × N cells (for example, 13 × 13, 26 × 26, and 52 × 52), with the size of each cell varying according to the input resolution. Predicting several bounding boxes and the associated confidence scores is the responsibility of each cell. The class probability distribution for each detected object and the confidence score of each box—the likelihood that it contains an object—are predicted by the model using a deconvolutional head. Non-Maximum Suppression (NMS) is used to remove redundant boxes with lower confidence ratings after prediction. Lastly, the Intersection over Union (IoU) metric (Equation 2) is used to assess the processed predictions (not wearing a helmet). It helps determine spatial links between observed items by measuring how closely a predicted rider’s bounding box overlaps with the associated two-wheeler. As illustrated in Figure 9a, the YOLOv8 model denotes “label 0” over a red bounding box when a motorcycle rider wearing a helmet is correctly predicted. Similarly, a rider without a helmet is indicated by “label 1” over a pink bounding box, and its corresponding number plate information is assigned “label 2” over an orange bounding box. The cases of accurate helmet violation predictions, number plate detection, and confidence scores are shown in Figure 9b. These results reveal that the suggested approach was able to distinguish between a cap, a scarf, and a helmet. Common failure cases observed during testing include missed detections in low-light or occluded conditions, as indicated in Figure 9c. When several riders are near one another, rider localization fails, resulting in overlaps of the bounding box. A more varied and enhanced dataset, particularly one that covers edge circumstances like night scenes or congested traffic, can help to reduce these problems.

#### 3.2.3 Recognition of license plates

**Figure 10:**
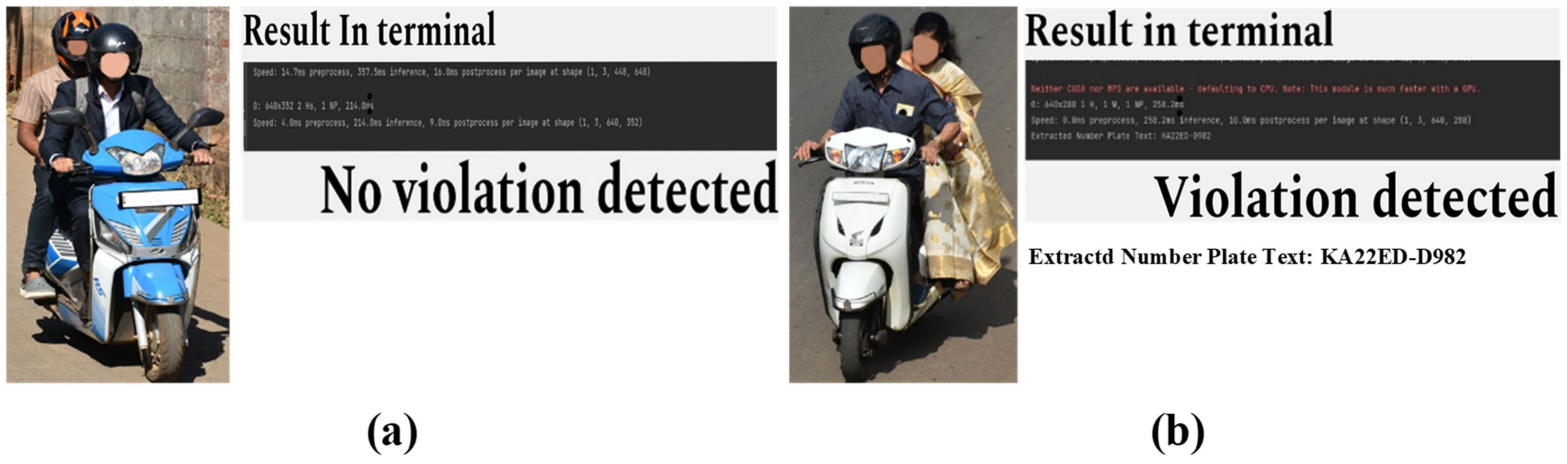
ANPR Number plate extraction results. **(a)** No violation. **(b)** Violation detected, and vehicle number displayed.

Our ultimate goal is to deploy the trained Automatic Number Plate Recognition (ANPR) model for real-time violation detection by integrating it into our Automatic Rider Helmet Violation Detection system to identify and translate each number on the recovered license plate and display it on the interface. As highlighted in Figure 10a, our system extracts number plate information upon detecting a helmet violation, otherwise, it bypasses the number plate extraction process on no violation, as indicated in Figure 10b. This integrated approach streamlines result verification and empowers users to promptly address traffic violations. For small object detection (such as number plates), multi-scale training, incorporating temporal information from video frames, and using higher-resolution inputs can all improve the accuracy and robustness of the model.

### 3.3 Performance evaluation metrics

As discussed in section 3.1, we created our custom dataset of 1,715 images containing 1,125 riders with helmets, 590 riders without helmets image instances. We use the 80:20 split on cleaned data (1,200 images) for training and validating our data. The object detection model’s effectiveness is determined by the following parameters:

- *box loss, train/box_loss, val/box_loss:* The degree to which the ground truth boxes and the anticipated bounding boxes surrounding the riders match is measured. Better performance is indicated by a lower value.
- *train/cls_loss and val/cls_loss:* It assesses how well the model can determine whether a particular area of an image has a rider. Better performance is indicated by a lower value.
- *train/dfl_loss, val/dfl_loss:* This represents the loss due to distortion. It gauges how accurately the model forecasts the rider’s stance. Better performance is indicated by a lower value.
- *metrics/precision(B):* This represents the rider class’s precision. The number of real riders among the detections the model made is measured using a metric called precision. Better performance is indicated by a higher value.
- *metrics/recall(B):* This represents the rider class’s recall. The number of real motorcyclists in the footage that the model was able to identify is known as recall. Better performance is indicated by a higher value.
- *metrics/mAP50(B):* This measure represents the Mean Average Precision (mAP) at a 50% Intersection over Union (IoU) condition. The mean area of prediction (mAP) is a widely used metric for evaluating object detection systems. It considers both precision and recall, providing a single score that reflects the overall detection ability. A greater value indicates better performance.
- *metrics/mAP50-95(B):* At the 95 and 50% IoU levels, the Mean Average Precision (mAP) is represented. It is like the mAP50(B) but evaluates the model’s performance across a wider range of IoU thresholds. A greater value indicates better performance.

## 4 Experimental findings and observations

We utilize a free, cloud-based Jupyter Notebook environment provided by Google Colab to train and test the DetectNet_v2 and YOLOv8 models. Google Colab provides powerful computational resources, including GPUS and TPUS. We introduce a two-step deep learning architecture for helmet detection in real-world traffic environments. This approach deviates from a single, monolithic model by employing separate modules for distinct detection tasks. This section delves into the rationale behind this design choice and its benefits. We trained and validated our models using Google Colab, a cloud-based platform that provides GPU resources for deep learning applications. With this method, the model was trained effectively and remotely by utilizing Google Colab’s processing power. At the first stage, the proposed DetectNet_v2 model is trained to locate riders from a traffic scene using the dataset containing 1,107 images (886 training and 221 validation). In the second stage, the proposed YOLOv8 is trained to identify riders with/without helmets and extract their number plate information using the dataset that contains 1,071 images (792 training and 279 validation).

### 4.1 Training and validation of DetectNet_v2 (model 1) for rider localization

**Figure 11:**
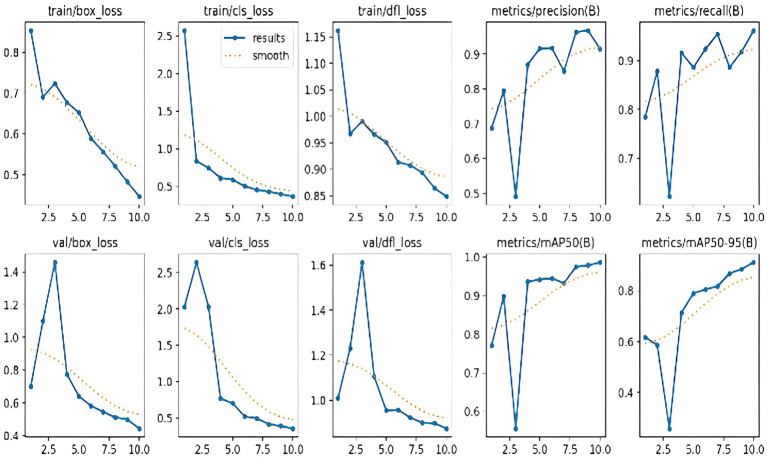
Training/validation loss curves and different evaluation metrics of the DetectNet_v2 (Model 1) rider detection model.

We used Pre-trained TrafficCamNet, a model developed on the DetectNet framework, to fine-tune and train on our custom dataset. In TrafficCamNet, a two-wheeler object is annotated when it has a bounding box size of at least 10 pixels in height or width at 1920 × 1,080 resolution. Partially visible objects are labeled as occluded, whereas those with less than 60% visibility are not. Hence, labeled objects are those that are at least 60% visible, and items at the frame’s edge that can be seen are marked as truncated. Fine-tuning is carried out by updating the model parameters using our custom dataset while retaining the foundational feature representations. We set a lower learning rate to prevent overfitting and preserve useful base features. Hence, to train and test the fine-tuned DetectNet model, we pre-processed images and carried out each experiment using the NVIDIA TAO (Train, Adapt, Optimize) transfer learning toolkit. We used TensorFlow as the backend framework and TensorRT as the inference engine. We used SGD with momentum as the optimiser with a weight decay set to 5e^−4^. We train the DetectNet model for 25 epochs with a batch size of 8 per GPU, using a learning rate of 0.01 and a decay rate of 0.1 every 10 epochs during the training. After applying TAO optimizations (like pruning, quantization, and conversion to TensorRT), we observed 70% latency reduction and around 150% increase in Frames Per Second (FPS) throughput. The crucial task of identifying and separating two-wheeler riders from other vehicles in the traffic scene is carried out by DetectNet_v2. This stage makes use of a dataset that has been carefully selected to include a range of two-wheeler riders in a variety of traffic situations. Here, the model is being trained to identify riders and generate bounding boxes around them. The model outputs the identified rider zones as bounding box coordinates or cropped images after training successfully. The number plate localization and rider helmet detection systems use this data as input to identify traffic infractions. Figure 11 displays the model’s training and validation performances. The loss curves typically decrease when the model is trained. This suggests that the model is gaining knowledge. The validation loss curves are usually higher than the training loss curves. This is a common phenomenon in machine learning that suggests the model may be overfitting the training data. The precision and recall scores appear to be over 0.5 for both the training and validation sets. This suggests that the model has a high degree of accuracy in identifying cyclists in the videos. Furthermore, the map metrics are higher than 0.5 for both the training and validation sets. This offers additional evidence of the model’s excellent performance. The curve shows how well our DetectNet_v2 network is learning to recognize riders in videos.

Overall, the DetectNet_v2 (Model 1) produced 98.6% accurate rider prediction on the validation set, and this helps implement stage 2 (Model 2) of our work, i.e., accurate helmet localization and detection process, which will be explained in the next subsection. Table 2 summarizes the pre-trained TrafficCamNet (DetectNet) performance comparison with our fine-tuned DetectNet_v2 (Model 1).

**Table 2:** Pre-trained TrafficCamNet (DetectNet) vs. fine-tuned DetectNet_v2 (Model 1) comparisons.

Dataset/model	Precision	Recall	Accuracy	mAP@0.5	mAP@0.5:0.95
Pre-trained TrafficCamNet (DetectNet) [30]	91.65	89.95	83.9	Not Reported	
Proposed fine-tuned DetectNet_v2 (Model 1)	92	96	98.6	0.99	0.90

### 4.2 Training and validation of YOLOv8 (model 2) for rider helmet detection and number plate information extraction

The process of helmet detection and number plate identification using the YOLOv8 model is explained in this section. DetectNet_v2 carries out rider localization by processing the full image to identify and generate bounding boxes around potential motorbike riders. These localized rider regions are then cropped from the original image and passed individually to YOLOv8n for further analysis. We used a pre-trained YOLOv8 model to fine-tune and train on our custom dataset. This pre-trained model is designed for speed and efficiency, and it uses an anchor-free, fully convolutional architecture optimized for edge deployment. Its fully convolutional, anchor-free architecture is geared for edge deployment and is designed for speed and efficiency. The fine-tuning process involves applying sophisticated augmentations such as Mix-up and Mosaic, and training the model with bounding box regression, object-ness, and classification losses. By systematically changing the number of trainable layers in the YOLOv8n backbone, we examine the effect of fine-tuning depth. Only the last detecting head layers, which correspond to layer 22 in the standard design, were trained using the Ultralytics framework’s freeze parameter (YOLOV8 Ultralytics, 2025), which we set to 22. The original COCO pre-trained weights for the entire neck and backbone (layers 0–21) stayed frozen. On the COCO validation set, a pre-trained YOLOv8n (Ultralytics, 2025) achieves a precision of 0.872, a recall of 0.697, mAP@0.5 of 0.746, and mAP@0.5:0.95 of 0.504, making it a strong baseline for fine-tuning on custom tasks like helmet detection.

**Figure 12:**
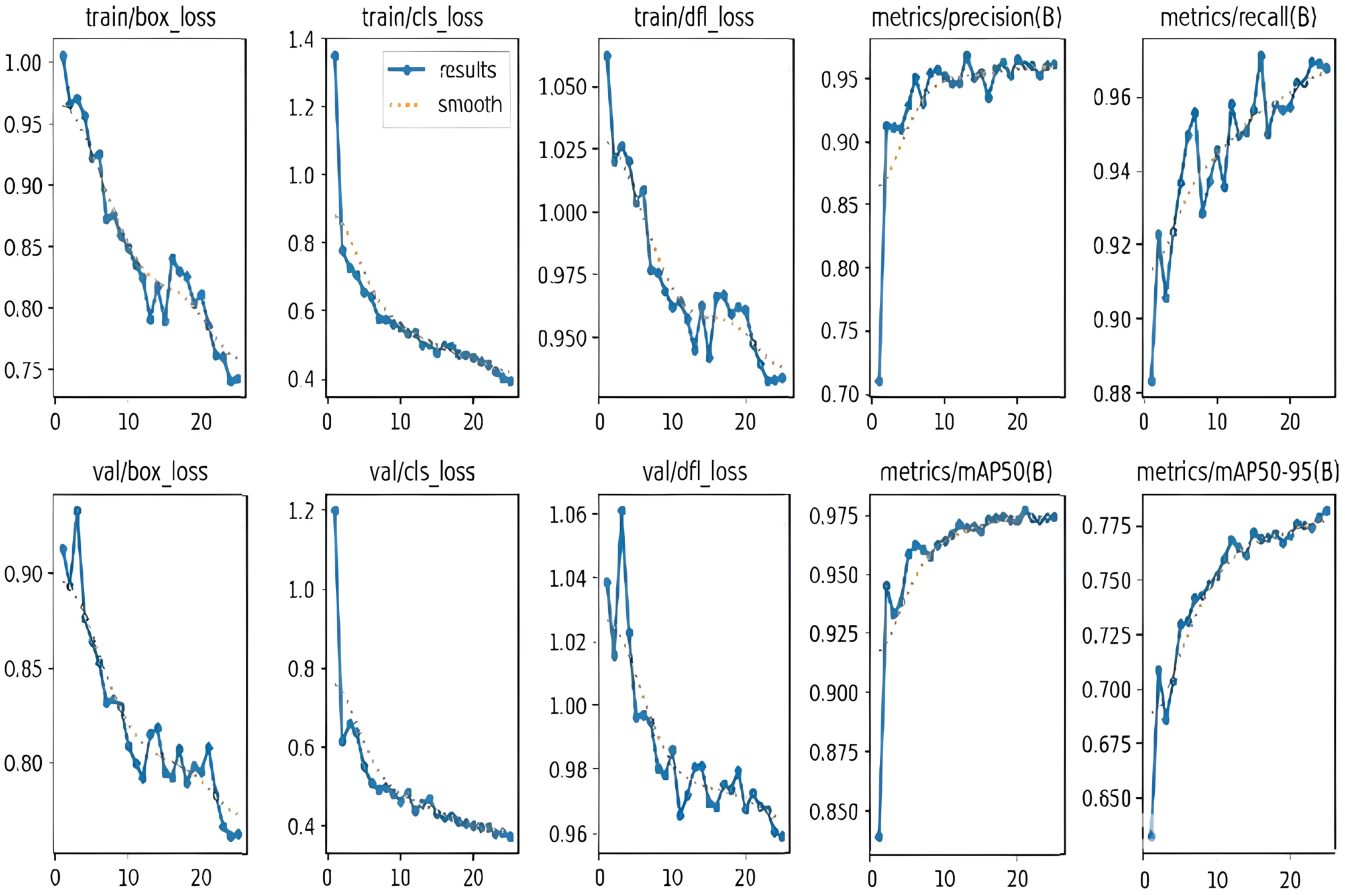
Training and validation loss curves and evaluation metrics for the YOLOv8 (Model 2) helmet detection model.

To train and test the YOLOv8n model, the pre-processed, re-scaled 640 × 640 pixel images are used. For rider/helmet detection and number plate extraction, we use PyTorch, which enables efficient training, transfer learning, and deployment. We selected AdamW as the optimizer with a learning rate set at 0.002 and weight decay set to 5e^−4^. We train this model for 25 epochs with a batch size of 8 per GPU, and a decay rate of 0.0005 every epoch. Once detectnet_v2 detects the bounding boxes for riders, these regions of interest (ROIs) are cropped or passed as input to a secondary detection stage using YOLOv8, a lightweight but accurate object detection model. During training, the YOLOv8 model learns to identify and localize these objects within images through bounding boxes and classification labels. The YOLOv8 model is pre-trained and fine-tuned on helmet and number plate classes to predict the objects with high speed and precision, leveraging transfer learning and optimized training settings. Once trained, the model can process new images or video frames and reduce false positives by performing helmet identification and number plate extraction exclusively within these ROIs. Intelligent traffic monitoring systems can use this two-stage pipeline in real-time since it increases overall detection accuracy and processing efficiency. Like the DetectNet_v2, the visualization of GPU memory consumption alongside box-loss, class-loss, and dfl-loss, observing their sizes and fluctuations throughout the training process, is depicted in Figure 12. The graphs display a variety of parameters and loss curves that were calculated during the training phase of YOLOv8. Table 3 summarizes the pre-trained YOLOv8n performance comparison with our fine-tuned YOLOv8 (Model 2).

**Table 3:** TrafficCamNet (DetectNet) vs. fine-tuned DetectNet_v2 (Model 1) comparisons.

Dataset/model	Precision	Recall	Accuracy	mAP@0.5	mAP@0.5:0.95
Pre-trained YOLOv8n (Redmon et al., 2016)	0.872	0.697	83.9	of 0.746	0.504
Proposed fine-tuned YOLOv8 (Model 2).	92	96	98.6	0.99	0.90

#### 4.2.1 Recall-confidence curve

**Figure 13:**
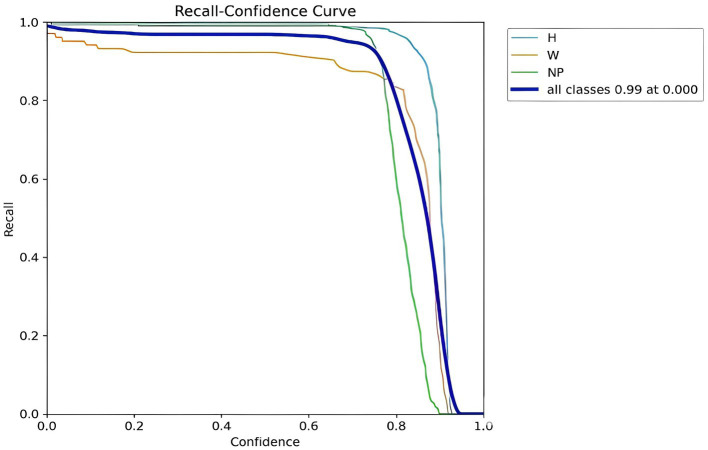
YOLOv8 helmet and number plate detection recall-confidence curve.

The model’s accuracy in recognizing objects like helmets and license plates is depicted in Figure 13, where the y-axis shows recall and the x-axis shows confidence score, revealing that the “H (Helmet)” and “NP (Number Plate)” curves demonstrate high recall even at low confidence—indicating the model effectively identifies helmets and license plates regardless of certainty, with recall increasing, plateauing, and then slightly declining as confidence grows. Whereas the “W (Without Helmet)” curve starts with low recall at low confidence and gradually improves, suggesting the model is uncertain of its prediction while recognizing individuals without helmets. However, the model becomes more adept at identifying individuals without helmets as its confidence level rises.

#### 4.2.2 Precision-recall curve

**Figure 14:**
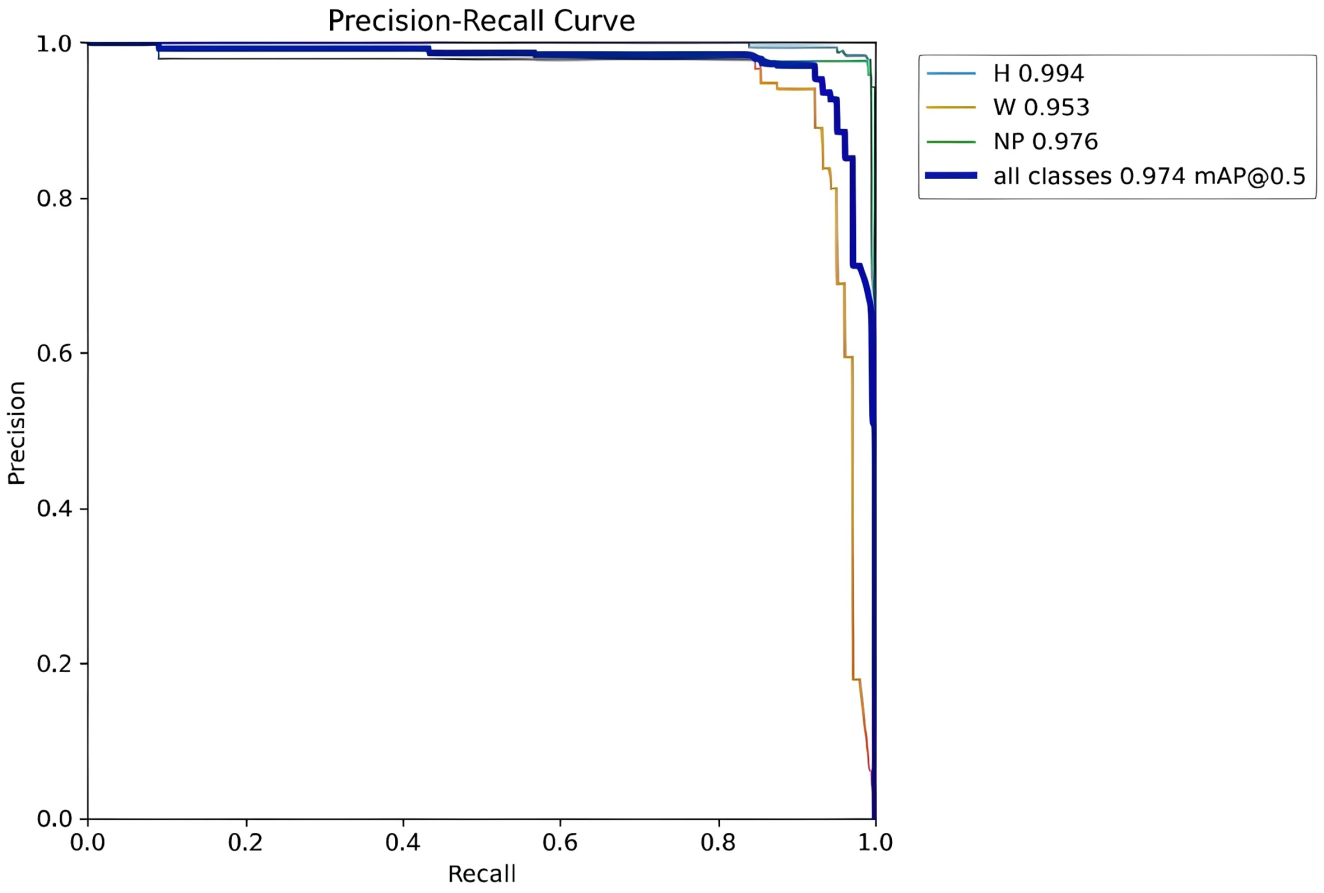
YOLOv8 helmet and number plate detection Precision-Recall curve.

As shown in Figure 14, the detection accuracy for Helmet (H), Number Plate (NP), and persons without helmets (W) is measured using a precision-recall curve, where precision (y-axis) represents the percentage of correct positive detections and recall (x-axis) represents the proportion of actual positives identified, revealing that the Helmet and License Plate curves start with high precision and recall at lower thresholds and then trade recall for higher precision as thresholds increase—indicating strong initial detection capability that becomes more conservative—while the Without Helmet curve starts with low precision that gradually improves with increasing recall, suggesting the model struggles with false positives at lower thresholds but becomes more accurate in identifying helmetless individuals as its confidence criteria become stricter.

#### 4.2.3 Precision-confidence curve

**Figure 15:**
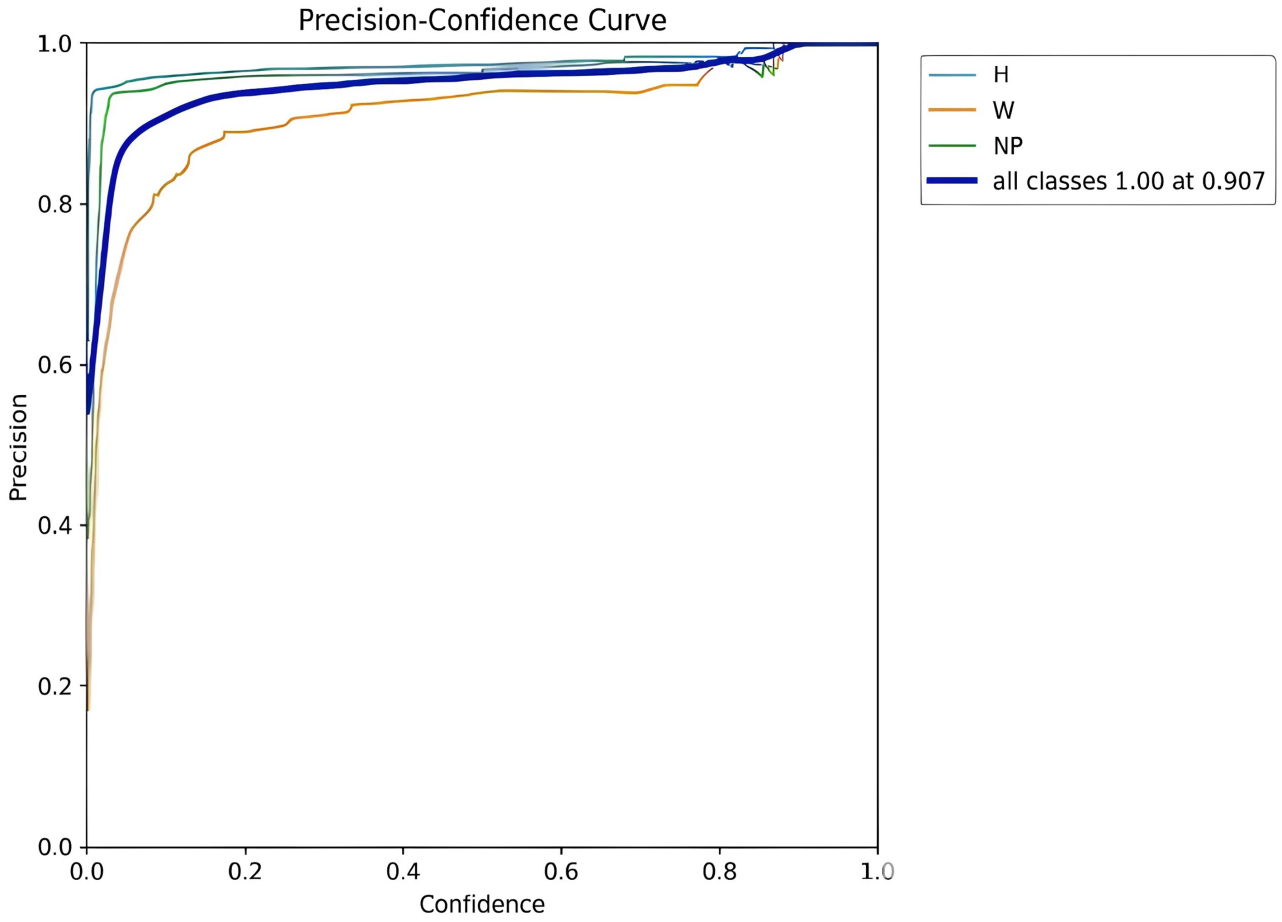
YOLOv8 helmet and number plate detection precision - confidence curve.

Figure 15 Presents the precision-confidence curve, where the x-axis shows the model’s confidence level and the y-axis represents precision (the ratio of True Positives to all predicted positives), illustrating that for all three object classes—Number Plate (NP), Helmet (H), and Without Helmet (W)—the model achieves perfect precision (1.0) at a confidence threshold of 0.907, indicating that when the model’s confidence is 0.907 or higher, its predictions for all object types are highly accurate.

After training the model and determining the best weights, the model is validated for the 279 validation test images. The model summary is provided in Table 4.

**Table 4:** Summary of the proposed YOLOv8 (Model 2).

Parameter	Value
No. of layers	168
Parameters	11,126,745
Gradients	0
GFLOPs	28.4

The time taken for the model in the prediction and validation phases is summarized in Table 5. The results show that the model takes a longer simulation time during the training and validation phases, but the predictions are made in less time.

**Table 5:** Simulation time in different phases.

Task	Pre-process (ms)	Inference (ms)	Post-process (ms)
Training	2.1	16.2	12.9
Prediction	0.8	29.41	10.4
Validation	2.4	16.7	13.2

### 4.3 YOLOv8 (model 3) as the rider localizer in the end-to-end implementation

Accurate rider localization is crucial in deciding the performance of the end-to-end automatic rider helmet violation detection and vehicle identification systems. Initially, we evaluated the rider localization performance on YOLOv8 (Model 3) with a model configuration the same as that of Model 2. The training and validation performance and observations are summarized in Table 6. Our proposed DetectNet_v2 (Model 1) under test outperforms the YOLOv8 (Model 3) on all significant two-wheeler rider localization evaluation metrics. By achieving significantly lower training and validation losses, DetectNet_v2 emerges as a reliably converging and dependable model. Its superior localization and generalization accuracy are noteworthy and led us to select it as the 1^st^ Model in the end-to-end pipeline. While YOLOv8’s current architecture and extended training duration result in smoother loss curves, its low mAP and recall indicate a higher incidence of missed detections, reflecting limitations in detection performance despite stable training behavior.

**Table 6:** DetectNet_v2 (Model 1) vs. YOLOv8 (Model 3) rider localization performance ablation study.

Metric	DetectNet_v2 (Model 1)	YOLOv8 (Model 3)	Comments
Train loss	Sudden early drop, but stable	Steady decline	DetectNet_v2 moderate performance
Validation loss	Fluctuates early, later smooths	Consistent decline	DetectNet_v2 moderate performance
Precision	0.92	0.94	YOLOv8 performed slightly better.
Recall	0.96	0.87	DetectNet_v2 performed well
mAP50	0.99	0.91	DetectNet_v2 performed well
mAP50-95	0.90	0.67	DetectNet_v2 performed significantly well.

### 4.4 Confusion matrix

The confusion matrix showing the performance of our proposed model on a helmet and number plate detection task is described in Table 7. The confusion matrix compares the actual labels of the data (ground truth) to the predictions made by the model. Each row represents the class (H, W, N), while columns represent the model’s prediction values.

**Table 7:** Class-wise confusion matrix of the proposed YOLOv8 (Model 2) helmet and number plate detection model.

*N* = 279	True positive (TP)	True negative (TN)	False positive (FP)	False negative (FN)
Helmet (H)	178	02	06	22
Without a helmet (W)	97	02	10	00
Number plate (N)	210	02	08	00

These values from Table 7 are represented in the form of a confusion matrix and are indicated in Supplementary Figure 1.

The description of this confusion matrix is provided next.

*Helmet (H)*: 178 images of riders wearing helmets were accurately predicted by the model (True Positive). In two cases, riders were wearing helmets when the model predicted they were not (True Negative). The model predicted a helmet six times when none existed (False Positive). 22 images without a helmet were missed by the model (False Negative).

*Without Helmet (W)*: 97 images of riders without helmets were accurately predicted by the model (TP). In two cases, the model predicted a helmet when none was present (TN). In 10 cases, the model correctly identified riders without helmets when none were present (FP). Eight images in which no one was wearing the helmet were missed by the model (FN).

*Number Plate (NP)*: The model detected 210 images containing a number plate correctly (TP). There were 8 instances where the model predicted the number plates when there were none (FN). The model missed 2 images where a number plate was present (TN).

The class-wise detection performances are highlighted in the graphs shown in Supplementary Figure 2.

Rider helmet detection accuracy comparison between our proposed (DetectNet+YOLOv8) model and several state-of-the-art models, such as CNN-based and Faster R-CNN techniques, is shown in Supplementary Figure 3. With a helmet detection accuracy of 0.9856, our proposed model performs well over the other state-of-the-art models. Compared to CNN-based models and Faster R-CNN variations, our model exhibits a clear improvement, with Faster R-CNN (Waris et al., 2022) producing the closest accuracy of 0.9769. Whereas, the conventional CNN (Vishnu et al., 2017; Dasgupta et al., 2019) models lag substantially. This highlights the superior detection and classification capability of our proposed approach.

The additional performance measure of our proposed model and Faster R-CNN (Waris et al., 2022), which has produced comparable results, is summarized in Table 8. Our model has the highest scores for all reported parameters, including accuracy (98.56%), sensitivity (98.9%), precision (98.89%), and F1 score (98.9%). It also indicates a significantly lower false positive rate (2.02%), indicating fewer false positives. Overall, the results show how much more effective and reliable the proposed approach is for helmet and rider detection tasks.

**Table 8:** Experimental results and other performance comparisons with the state-of-the-art Faster R-CNN (Waris et al., 2022) method.

Performance metrics	Derivations	Scores	
		Proposed (DetectNet_v2 + YOLOv8)	Faster R-CNN [26]
Accuracy	ACC = (TP + TN)/(P + N)	0.9856	0.9769
Sensitivity	TPR = TP/(TP + FN)	0.989	0.9825
Specificity	SPC = TN/(FP + TN)	0.9798	0.9694
Precision	PREC = TP/(TP + FP)	0.9889	0.9770
False positive rate	FPR = FP/(FP + TN)	0.0202	0.0306
F1 score	F1 = 2TP/(2TP + FP + FN)	0.989	0.9798

## 5 Conclusion and future scope

One of the most difficult problems in a real-time traffic violation detection system is accurately identifying two-wheeler riders in complex traffic scenarios. Deep learning-based object detection techniques make it possible to identify and punish helmetless bikers. The proposed deep learning-based DetectNet_v2 and YOLOv8 two-stage models accomplish the task of automatic real-time rider localization, rider helmet violation detection, and number plate extraction. This real-time rider helmet violation detection system presents a promising foundation for enhancing road safety and enabling effective enforcement of traffic regulations. According to the experimental investigation, the proposed system produced helmet and number plate detection accuracy of 98.56 and 97.6%, respectively, under varying lighting conditions, weather scenarios, and night conditions. Missed or incorrect detections in these challenging scenarios, including occlusions and crowded situations, are frequent testing failures that can be avoided by using temporal video data, dataset augmentation, higher-resolution camera inputs, and multi-scale training. Even though the chosen 1,200 images from our custom dataset are diverse, properly labeled, and sufficient for fine-tuning in helmet detection binary classification tasks, the robustness can be further improved for real-world deployments with a greater number of images (2 k to 5 k). Additionally, estimating the motorcyclists’ speeds could enhance the proposed system’s features. This can be achieved through frame-by-frame object tracking with camera calibration, enhanced by algorithms like Kalman filters or optical flow, or, for greater accuracy, through external sensors like radar or LIDAR. However, combining this system with state-of-the-art edge computing technologies, like NVIDIA edge AI solutions, can further enhance the real-time detection capabilities. The system can function in a decentralized fashion by distributing the models on edge devices, which lowers latency and eliminates the requirement for constant communication with a central server.

## Data Availability

The raw data supporting the conclusions of this article will be made available by the authors, without undue reservation.

## Supplementary material

Supplemenatry Figure 1 Confusion Matrix of YOLOv8 helmet and number plate detection system.

Supplemenatry Figure 2 Class-wise performances of rider helmet and number plate detection.

Supplemenatry Figure 3 Proposed (DetectNet+YOLOv8) models for helmet detection accuracy comparison with the state-of-the-art methods.
