# Supplementary figures and images for: Computer-vision based automatic rider helmet violation detection and vehicle identification in Indian smart city scenarios using NVIDIA TAO toolkit and YOLOv8
Source: Front Artif Intell. 2025 Jul 22;8:1582257. doi: 10.3389/frai.2025.1582257 (PMC12321817; doi:10.3389/frai.2025.1582257)

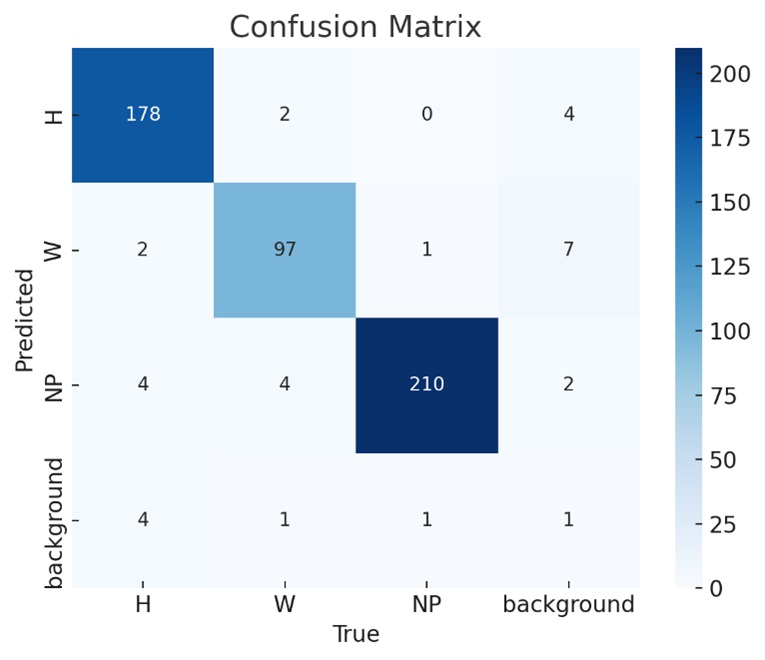

Supplement: Supplemenatry Figure 1 — Confusion Matrix of YOLOv8 helmet and number plate detection system. [file Image_1.JPEG]

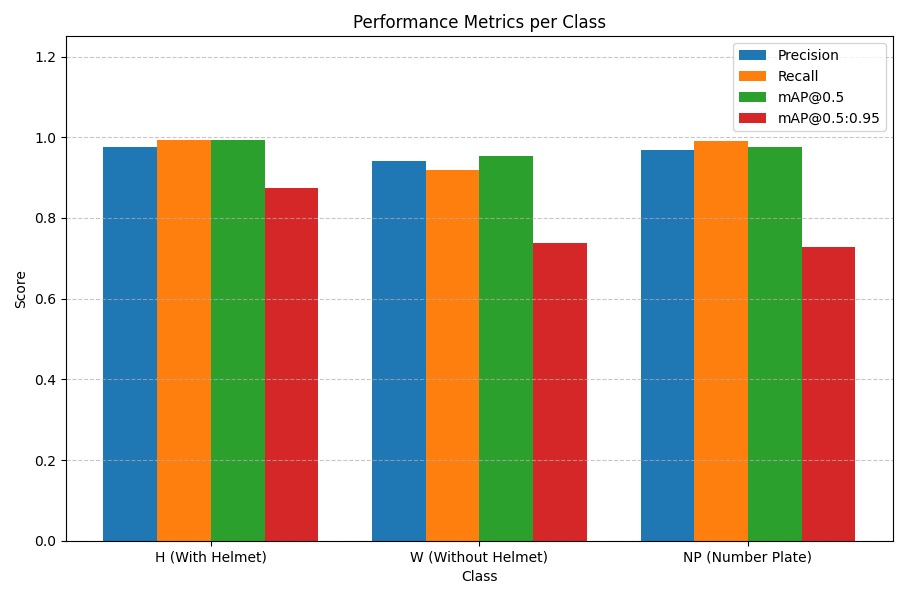

Supplement: Supplemenatry Figure 2 — Class-wise performances of rider helmet and number plate detection. [file Image_6.JPEG]

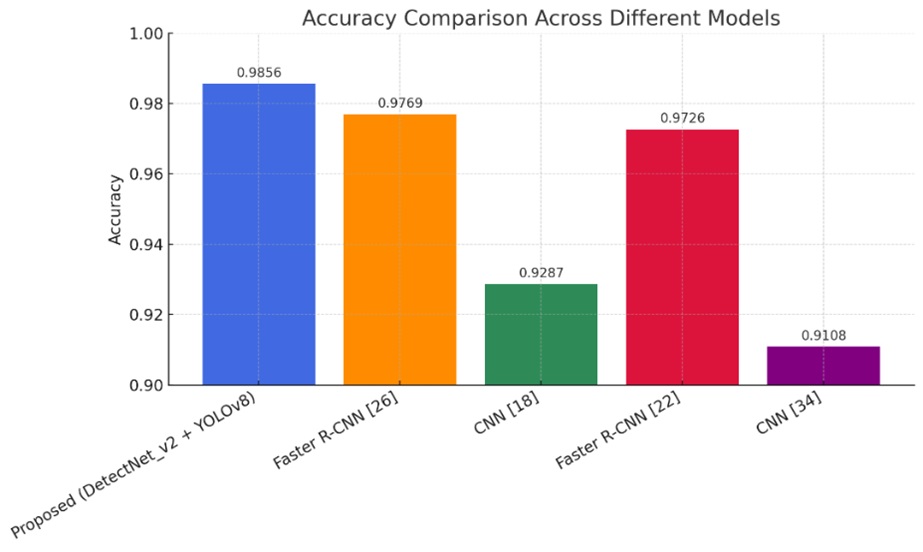

Supplement: Supplemenatry Figure 3 — Proposed (DetectNet+YOLOv8) models for helmet detection accuracy comparison with the state-of-the-art methods. [file Image_7.JPEG]
